# Why Do Fillings Fail?

**Published:** 1879-02

**Authors:** Wm. A. Pease

**Affiliations:** Dayton, Ohio


### THE DENTAL REGISTER.

VOL. XXXIII.] FEBRUARY, 1879. No 2.

### WHY DO FILLINGS FAIL?

BY WM. A. PEASE, DAYTON, OHIO.

There is at the present time a questioning of the results of dental operations in filling the teeth, and a growing dissatisfaction with achievement. All of the materials used for that purpose are undergoing a strict scrutiny as to their fitness, their endurance or protecting qualities, their liability to chemical or electrical change, and of everything that makes them more or less desirable as a stopping. Comparatively new materials, and new forms of them, that a few years ago gave promise of better results than had hitherto been attained, are not proving satisfactory; and, in the depression of the moment, there is a feeling that they are not as good as some that were abandoned. They are certainly not as good as what dentists had promised they would be to themselves, and what they had promised to their patients. It is the thinkers who severely question results, who ask these questions of one another, among themselves, in society, and in the journals. They are not satisfied with mere professional success; with anything but progress; and, after years of patient toil and search after betterment, they begin to fear that they have not progressed and attained greater certainty and capability in saving the natural teeth. They have endeavored to make this professional, have staked their standing upon it, and if their operations are not successful, and are essentially mechanical, where is the profession? Their appliances are beginning to smack now more of the shop than of the office. They are growing to be more complicated and costly; they are more deterring to the nervous, and they impress all as being more formidable, and are more costly in time and money. What has the patient got for all this—a filling that saves the teeth better than it did twenty years ago? That is the question! What say our thinkers and observers? As yet, they do not answer it, except inferentially, by questions as to the cause of numerous and confessed failures. Inquiry is working around the edges of the question, establishing a fact or dissipating an error here or there, and it will come to the center at last. There are several side issues to be disposed of; some prejudices, personal and pecuniary interests, before the cause of failure will be acknowledged, and decided steps are taken to remove it. The primal cause of the decay of the teeth is not now under consideration; that is known. But the cause of the cause is not so apparent, and at the proper time it may be worthy of consideration. What concerns the profession most at the present time, is the rapid decay of the teeth after they have been filled; either marginally or locally. After the best intelligence and care have been expended in preparing the teeth and filling them with various materials, why are the fillings insufficient? In a varying length of time why should decay appear around the margin of the filling, or at some local part of it in others; and yet, again, in other teeth and in other mouths it is absent, when all of the teeth were filled by the same person, in the same manner, with equal care and apparently under equal attendant circumstances? Such cases occur every day. Why is it? What law of nature works so unequally, with so many exceptions? And if it is not a law of nature, what is it? the material. And if not the material, what, the mode of using it? Of late galvanism has been thought capable of causing such decay, and it is accused of actually doing it; although I have seen no attempt to explain the numerous interruptions in its currents, its unaccountable skipping some plugged tooth equally exposed, and numerous other exceptions and excentricities. Perhaps it will be easier to explain them than to abandon a cherished material or a favorite mode of practice.

Galvanism is generated by placing and connecting two metals in a suitable solution, where one of them is attacked by oxygen and the other is not; one is wasted, oxydized, and the other is not. Lime in any of its forms can not become a pole of a battery. It can not be decomposed and lose its carbonic acid by any current that can originate in the mouth, and it is insoluble by galvanism. Hence all of the injury the teeth can suffer from galvanism is not from a direct current acting on them, but from some secondary product of that current, the decomposition of some material in the mouth. Two teeth freshly plugged, the one with gold the other with amalgam, if placed in a suitable solution, will generate for a very short time a feeble current if suitably connected, or until the surface of the amalgam becomes coated with an oxide, when the action will cease until the oxide is removed. An amalgam filling composed of various metals, as some of them are supposed to be, if placed in a solution, might excite galvanism of a very feeble and transient sort, but it would soon cease for want of oxidizable material. Suppose that the filling leaked, that is in some part of it moisture by capillary attraction found entrance between it and the walls of the tooth, and that moisture came in contact with one of the oxidizable metals; it might be very small, what a miner would call a surface indication, or it might be a pocket, that is a small and definite penetration into the filling, that might end in a pinch or a minute filamental penetration deeper; but that almost infinitely small filament of water could only part with the oxygen that moistened the surface. It could not penetrate and honeycomb that filling for want of oxygen, and even if it could the oxide on the surface would prevent corrosion. But this action is on the filling and not on the tooth, and the tooth can only be injured by the galvanism decomposing some material to form an acid. But what is there shut up in that cavity, away from the food, scarcely a trace, if any, of animal matter in the fibriles of the dentine to decompose? Suppose there are two fillings in the opposing faces of two bicuspids, one of gold and the other of amalgam —a hardly supposable case—running nearly to the gum, and the faces of both of these fillings are clean, and a little non-oleaginous animal fibre were placed between and connecting both surfaces, would there be a current that would roughen the place of contact on the amalgam? Suppose again that a pure gold and an amalgam surface, both clean, were connected with a thermometer tube open at both ends and filled with a suitable fluid, would we see a double current—water flowing to and leaving its oxygen on the amalgam, and hydrogen passing to the gold, until the tube contained only hydrogen, the oxygen being represented only by the rust on the amalgam? I think not, and yet this is theory. But theory calls for clean surfaces. Suppose we take the zinc from the cup, dry it and smear its surface with butter or lard, and replace it would there be a current, and at length, after the butter and lard had parted from the surface of the zinc and risen to the surface, would it yet be in condition to be corroded? Oxygen and oil are incompatible. Oxygen only acts on a clean surface. Suppose again that there should be packed around the zinc, animal fibre, oil cells, bread and butter, buttered potatoes, onions, cabbage, etc., and there should be poured over that a decoction of turtle soup, would that mass or any part of it be decomposed and form an acid or acids capable of dissolving the teeth? These suppositions are not so extreme as might at first be supposed; they are based on an oiled or unclean surface such as the fillings present in the mouth. No practical electrician would think of placing his zinc in ordinary spittle, containing oil globules, epithelial scales, mucus and some salts of lime, etc., and expect it to act, and no dentist building up a contour filling on an incisor, where success depended much on the base, would allow the filling to be wet when half done and expect fresh gold to adhere to the surface coated by saliva; there would be contact and not union, and the filling would part at that place. The theories of galvanism in the mouth are formed from batteries on the bench, where every thing is clean and in order, capable of giving strong or feeble currents according to the surface exposed, and where the important element for electrolysis, time, is not wanting. In the mouth everything is different. None but the lower teeth are even partially submerged; the upper are barely moistened by a fluid generally neutral, rarely giving an acid reaction, always scant, always changing by passing to the stomach or by expectoration. In some mouths there are but few fillings, often widely apart, of but one metal and having no connection; in others there are many kinds of different metals, and again, in others there are large suction plates of gold or platinum, carrying a few teeth, or running along by the side of the teeth, or dipping their serrated edges between them and in close proximity to fillings, which must necessarily be connected with them at times by animal fibres. Plugged teeth in such mouths, where so large a surface is covered with a plate, resting at times upon raw, or ulcerating patches, connected by food to fillings on the approximal surface, do not suffer more from peripheral decay than in mouths without plates; and these plates are more likely to excite galvanism, because they are frequently taken from the mouth and cleaned.

Silver will waste and tarnish in some mouths. So will Watt's and kindred compositions, but they retain their color better, waste less, and their platinum pins do not become as prominent; but a filling made of Watt's metal does not waste as much as the plate, probably because it is not taken from the mouth and cleaned. There is a very large amalgam filling that I put into the front face of a superior molar twenty-eight years ago, and running past it, and nearly touching it, there is a Watt's metal plate that has been worn about five years. The plate wastes, but the filling does not, and the margin of enamel around it is sound. This would indicate waste by galvanism and acid, or by much scrubbing. Oxygen may attack one of its constituents, acid may be present, but the cleanliness of the plate indicates the brush. The amalgam filling has a slight film over it, like all other fillings, and it is dark around the edges.

The sulphur stain, when coated by a film of oily mucus and other materials, that enter the mouth, becomes a shield against further attack, and the dark border of it, near the enamel, would be little affected by anything weaker than a strong acid or alkali. It has never been my lot to see much waste of these fillings, but I have frequently seen marginal waste of the teeth; and the waste of the fillings was, probably due to improper manipulation or inferior material. The oily, sulphur stained and very unclean surface of stoppings, bordering on and next to marginal decay, are in very unfavorable condition to excite galvanic action. They do not do it; and that marginal decay increases from the same cause that decay increases in other and unfilled teeth. Whenever I see a dark and sulphur stained filling, otherwise good, I think that tooth is as safe as filling can make it; and I should have as much compunction of conscience in removing that filling, and replacing it with gold, as I would in cutting and filling a cavity in a sound part of the tooth. An unstained amalgam filling is suspicious, worthy of attention, because it must contain an excess of tin, or other material that might excite galvanism, were not all fillings, especially in the back of the mouth, more or less coated with a film. Laborious brushing might, in some mouths, with the aid of a sharp dentrifice, remove the film on exposed points, and there might be a fluid in the mouth sufficiently strong to attack it; but the margin would be safe, and the probability of such concurrent conditions is small, especially as the saliva is ever present to recoat it. An amalgam to be good should be simple. Silver is the amalgamating metal, and unless it is an alloy all that is put into it separates its particles as the coloring matter does in rubber. For this reason I aim to keep by me pure silver for desperate cases, as it is harder, its hold on weak retention points is better, and its black, honest face is assuring.

In 1848, and a few years thereafter, I experimented with an amalgam containing gold, in varying proportions; and while it tarnished less, it aroused suspicions, and not being more satisfactory generally it was abandoned after a time. At that time I had a battery on my bench working by electrolysis; plating silver plates and occasionally watch cases and other articles for jewelers. That taught me the appearance of zinc in the battery, and how to take care of it; and it, also, taught me the appearance of a twenty dollar piece, or a gold plate that was being slowly transferred from one pole of the battery to the object to be plated; and it taught me the effect of oxygen or of sulphur on a silver plate in the mouth, when the gold had been worn from places on it, or it had pealed off. Since then I have been careful about amalgams, and there are some makes that I avoid, partly because they are coarser, and their particles are more difficult to break down—they do not yield kindly to quicksilver, and there is an appearance in their light color that is objectionable; and they seem to require more quicksilver. Unless long worked, fillings made of them roughen, and some ingredient in them, having softened more than others, wears away and shows the harder original fibres, unsoftened and prominent, and leaving little depressions on the surface between them, and also at the junctions of the enamel to invite peripheral decay. An excess of quicksilver is answerable for much of the decay in teeth filled with amalgam, as in packing, it goes to the surface of the cavity, inside as well as out, it afterwards evaporates, and gives the juices of the mouth access to the inside of the cavity. This causes peripheral decay as well as moisture, a fissure, rough edges of the enamel, or a particle of partially decomposed bone, carelessly left at the surface. While amalgam will harden under water, pressing it into a cavity will not expel moisture, a mere trace of which is a lead that by capillary attraction will draw more into the cavity and renew the decay. Capillary attraction is a thousand fold more damaging than galvanism; it is the great tooth destroyer after filling; whether the filling be gold or amalgam; and it should be seriously thought of and discussed in proportion to the magnitude of the danger and damage it occasions; beside it the discussion of galvanism is most stale and unprofitable. To secure a filling against it great care, good judgment and a conscientious skill and attention to detail is necessary, not only to thoroughly prepare the cavity, but to leave no thin edge to be crushed, a fruitful source of decay, and to see that the cavity is dry; that no superfluous quicksilver goes into the silver to be squeezed out, and that none is put there but what is necessary to harden it. It is a mistake to suppose that a cavity, that will retain a gold filling, will, always, retain an amalgam, for this reason, that there may be two points between which gold may be driven, and so compressed, as to be pinched and held firmly; but the amalgam being soft, simply fits the place, and after setting, there is no pinch to retain it. For this reason care should be taken with the base, as amalgam has a tendency to round off and slip by any sharp depressions without accuracy of fit, and special attention must be paid to them; and for the same reason, if there are sharp retaining points, the walls are strong, and care is taken to fill them, it is the best material for building up parts of molars, as it does not wear or break and disintegrate. It has like gold or other materials great capabilities if properly developed, and when judiciously used it contributes much to human health and happiness; and it saves a deal of pain and misery. For myself I consider it my first duty to save the tooth, to save suffering, present and prospective, irrespective of material, and then, if the case admit of it, choice of material may be considered. To that end I do not hesitate to use it in the front teeth for those who are either unable to pay for it, or prefer it; giving them instructions how to keep the surface from too much stain, if it is exposed, leaving to others to determine the æsthetical difference between a dull surface and an obtrusively bright and shining gold one. I also use it for young people, as a temporary or permanent stopping, as they may determine in after years; as I firmly believe that it is the best for a sensitive, nervous girl's incisors at twelve years of age, who has been frightened by the tales of others, until she approaches the dental chair almost paralyzed with fear and with nervous exhaustion. For her it is better than gold; as it is easily inserted without malleting or unnecessary manipulation; it saves her nervous system from shock, gains her confidence, and she is ever after that under better control, she is a better patient, and a healthier and better woman. Respect for the nervous system of the young, and abstention from shocking it, verge on piety.

More than thirty years ago I put both kinds of fillings into the same mouth for a patient in this city, that I have frequently seen, and have seen recently; having had occasion to replace two gold fillings in rather crowded central incisors for marginal decay near the gum, caused by the refusal to allow me to file away the palatine edge of the teeth as much as it ought to be. The balance of these soft gold fillings are good, their faces are bright and clean, and the very large amalgam in a molar is unchanged and apparently unchangeable. These were not exceptional cases; the health of the lady was not unusual, and there were some contra-indications that were not assuring, while the stoppings in the teeth of numerous other parties are nearly as enduring; and of the large army of them that went into the war to battle with hard tack the survivors are numerous and healthy. This is the more assuring as my confidence has been great, and my risks large. I often filled teeth that I supposed must be but temporary, that have outlasted my expectations, and the final cause of failure has been the breaking away of a piece of the tooth rather than the failure of the filling. In all of the history of amalgam it has silently and unobtrusively held its way; always under a cloud; always unfashionable with those, who aspire to leadership; always used under a protest and with mental reservation; it has been its lot to be generally used as a last resort; as a forlorn hope; for those desperate cases when gold was acknowledged to be inadmissible, either from the difficulties of the case, or the great expense attending it; while in all of the small, easy and accessible cavities gold has been used; and yet, its record is good, notwithstanding the many incapable, protesting and unenthusiastic hands that have used it, and abused it, or removed it when it was doing well, from all cavities, when they were permitted, and gold could be substituted, unmindful of the terribly frequent and costly failures of that metal. And yet, to-day, taking into consideration my long range of experience, and my observation of the failures and successes of others, if I were asked which of the two, gold or amalgam, had been the most beneficial to my patients and to others, had saved the teeth that needed saving the most, and the most of them, I should unhesitatingly answer amalgam; and after all, my fillings have been chiefly of gold. In making this estimate I have not been unmindful that there is a class of cavities where decay is suspended and has become dormant, with little likelihoood of revival, that if filled would go to raise the average of gold. These have generally been filled, often unnecessarily, by dentists who are either needy or greedy.

I have had a few, inconsiderable and spasmodic suspensions in the use of amalgam out of respect for what dentists call expediency and raising the professional standard, but as I have found nothing to take its place, it has been reinstated in my practice and affection. At such times I have used tin, or a solid ingot of tin, that was quite satisfactory in the cases adapted to its use; but as these were limited, and although it was brighter and looked cleaner, it was not more lasting, and it was discontinued in the main. If those who use tin wish to experiment with it, it is easily done, as all that is necessary is to press three or four sizes of wire into moulding sand, or make a matrix in plaster and pour in pure tin. Dress the cavity with a bur and select a rod of tin the same size, or file it to fit, cut it off the right length, press it in and then drive a broken excavator, sharpened like a wedge, into it to spread it at the base; tamp the edges, as well as the cavity in the middle, a little, dress down and there is a filling that does not condense in eating on it, or wear, and is light colored. If there are sutures they can be filled with foil or smaller ingots. These fillings are tight and they do not shrink.

Gold has been long used as a material for filling the teeth and there was a time when it was effectual. The principles involved, after the cavity has been prepared, are the same as those used by a plumber in making a tight joint, that resists great hydraulic pressure without leaking, while the principal cause of the failure of gold fillings is leakage without pressure. The cases are similar, and yet, they are not. The plumber has a stout margin against which to pack his lead, while the dentist often has not, and he has greater accessibility than the dentist, and a more plastic material; but still, I never see a plumber connecting pipes, that I do not stop to see if I can not get a suggestion from him. Until about twenty years ago gold was crowded into a cavity much as a plumber makes a joint, and the fillings were good, durable and seldom leaked; and we find them frequently now in good condition and doing better service than the average filling of five years ago. But the range of its application was limited, as it could not be extended beyond the retaining walls and successfully resist wear. About that time it was found that by annêaling gold it acquired two properties; one of which was desirable, and the other not; and that for many purposes the objectionable qualities of the one counter-balanced the good of the other. The first was the cohesion of one piece to another, by means of which a filling might be carried beyond the retaining walls with considerable success—this was valuable. The other was a harshness, rigidity and resilience, that made it difficult to adapt it to the walls and sharp angles in a tooth, and keep it from springing back when placed there. When the gold was anchored to the base the difficulty was measurably overcome, because piece after piece could be added to it without the manifestation of recalcitrancy as it adhered to the base wherever it touched. But that adhesion was so slight, and its resistance to condensation was so great, that the pressure of the instrument in the hand was insufficient to condense it, and make it conform to the cavity—it would leak. To use it, sharp, multi-pointed instruments had to be devised, and the sharp impact from the blow of a mallet was substituted for hand pressure. Under the old system, a piece of gold was pressed to its place until it conformed to the shape of the cavity, and when the instrument was withdrawn it staid there—there was no doubt of that, and the fillings were tight and good. Under the new, the piece of gold might be driven to its place and it might stay there, but, after a time, there may be reaction. The rebound may be slight, not enough to attract attention, but enough to draw in the fluids of the mouth by capillary attraction; an element of uncertainty to be measured only by experience, if by that. In the one case, we have the valuable assistance of an educated hand, that has become so sensitive, that it can tell with almost unerring certainty, when the gold is firmly against the walls of the cavity, and when not; in the other, there is no measure of resilience, but only of density, as shown by the penetration of the point of the instrument. Again, in many cavities in the approximal face of the teeth the filling is driven to the base, next to the gum first, but it is frequently impractical to condense a filling in that direction to completion; the direction of the instrument must be changed to nearly a right angle to that, to condense it against the side, which may, and frequently does, spring the stiff and resisting material from its base on the opposite side, and, if the direction of the instrument is again changed to condense it against the other side, the danger of loosening it is again imminent. The difference between the steady pressure of the hand, under which the soft gold glides slowly and steadily to its place, and the sharp impact from a mallet in condensing an elastic material is very great. But the withdrawal, if any, is slight, unnoticed, and the more dangerous, as a visible defect can be remedied, but the slight is only apparent in after years. This may be one and the most prominent cause of decay so much complained of now; it is undoubtedly due to leakage, and there is no other adequate cause for it.

With annealed gold have come contour fillings, that have been pushed to an unreasonable and unphilosophical extent, by which their permanence is endangered. To counterbalance this it is necessary to cut retaining points near the cutting edge of the incisors, or slots on the platine face that materially weaken them and endanger the whole tooth for the sake of a strip of gold running along the edge of it to restore the original shape. It is doubtful if this would be submitted to as a matter of taste alone, as the difference between a slight deficiency and that deficiency restored by a shining bar of gold much more observable, is not in favor of the latter, were it not urged as necessary to save the tooth. Such restorations must necessarily be temporary, as the bar of gold between the retaining points at the base and the cutting edge is only that of approach to the dentine and enamel, and it is not water tight. An examination of it with a glass of moderate magnifying power would show how worse than useless it is as a means of saving the tooth. All that is generally necessary for the preservation of such a tooth is a slight dressing and possibly a filling at the base, that leaves no place for the incubation of disease. But such fillings are now the fashion, as they increase patronage and advertise, and a young dentist never feels that he has won his spurs until he has made a few of them, and made some one as ridiculous as the shoddy women during the war, who thrust their hands into the face to show their diamonds, or Biddy with her silver or brass rings on her fat and purple fingers. The restoration of the lost substance by contour fillings is the restoration of the condition that preceded the original decay and made the filling necessary; plus the danger of decay at the junction of the gold with the edge of the enamel at the base. An isolated tooth having a healthy enamel, never decays unless food be allowed to lodge near the gum. That same tooth crowded to the side of another is very liable to decay at the point of approach and the decay may be mutual; or it may be confined to one, with a stain or incipient decay on the other. Remove the decayed tooth and the incipient or slightly decayed spot immediately assumes a new phase, takes on new characteristics, and what was beginning to be active becomes passive or dormant, an indefinite suspension of function in a healthy mouth. But substitute a contour filling for the removal of that tooth and the decay in the other becomes active and continuous. Decay may be said to attack a tooth at the most vulnerable point, not always of inherent weakness, but of exposure. The filling of that point transfers the point of vulnerability to the edge of the filling at the median line between the two teeth, at the base first, at the sides next—the already broken enamel against which the filling rests or overlaps is an imperfect protection and a slight diminution of vulnerability. But the base of that filling at the median line nearest to the gum is a point of exposure greater than that, which pre-existed and antedated decay, and a contour filling is a fosterer of decay at that point by superadding its influence to that pre-existing. Isolation destroys pre-existing tendencies, and the filing away of a small cavity in one tooth in such a manner that the teeth can not approach, destroys pre-existing tendency in the other, and arrests incipient decay. Whatever that insidious principle may be that we call caries, it never attacks in the open, but always, like the Indian, by stealth and in ambush, and our efforts must not be directed to the making of an ambuscade, but to breaking it up. Instead of bending our energies to the study of galvanism in the mouth, or to the enmity that is fancied to exist between dentine and gold, we should study the enmity of tooth to tooth, of enamel to enamel, when brought into close neighborhood, and if we can not find some remedy for it, we should break the neighborhood up. Contour fillings that restore contact should be banished from the teeth of the young. That would be a positive gain. Then, if we could reintroduce the file so as to prevent palatine contact, of the incisors and bicuspids, and leave a considerable space, then much good would be done. Then with soft gold that should not be attempted to be carried beyond the filed surface, we might succeed in making a water tight filling as the plumber makes a water tight joint. In this way teeth might be filled so thoroughly that the average duration of the fillings might be twenty years before the appearance of marginal decay, instead of five as it is now; and it is believed that when the cavity is filled without filing it is even less than that. Said a very experienced filler to me recently, speaking of the failure of fillings, "I am completely discouraged; I do not know what to do; when I see women wringing their hands, the tears running down their faces from the agony of an operation that they must endure to have their teeth filled, and when I think in three or four years this must be all gone over again on account of fresh decay near the fillings, I am sick. It makes me sick to think of it, and yet I do every thing I can to save the teeth." This man is noted for his thorough and painstaking care in filling. One of his conditions in operating is, that he shall do what is necessary, unquestioned and unopposed. His appliances are good, his fillings are dense and well finished, and in his ability to manipulate and control gold he is the peer of any man. His fillings to-day do not protect the teeth as well as they did twenty years ago, and this is due to the change from soft to annealed gold, and yet he can not change and go back to the practice he has abandoned and succeed. Dentists had long wanted a material that would fill out a nick in the enamel of an exposed tooth and give a better face to it than soft gold, and when they found they could build up with the annealed, they experimented with it and pushed it to extremes. It was a straw that tickeled the mature as well as the immature; each played with it, experimented with it, praised it to his patients until it became fashionable, and fashion not only ruled the patient, but the patient ruled the dentist—to be unfashionable was to be old fogy. Patients instead of, as formerly, wishing to hide the filling, now wish to show it, and they do not want their teeth filed to gain space and contribute to cleanliness. Annealed gold has its uses and abuses, and probably always will have. The property of hardness and elasticity it acquires from absorption of carbon from the flame may possibly be overcome and leave it cohesive. To anneal it with the electric light or coil may add to its present qualities those that we need, or some other mode of treatment may improve it. Crystal gold has many of the properties of annealed gold that are desirable, and some that are better. It is eminently plastic, and it takes the shape of every cavity into which it may be pressed, and it never springs back. Under proper manipulation it makes a water tight filling, and it is consequently seldom surrounded by that marginal decay that has been attributed to galvanism. It is equally as cohesive as annealed gold, and at the same time, it is so soft as to need only hand pressure, although the mallet may be used in condensing some part of the filling, and it can be passed through the flame of the lamp without losing its plasticity. When condensed into a filling the union of its particles is more intimate than any other material, forming for density and finish the highest type of a filling, one that can not be disintegrated—a nugget. Examined under a glass it shows no striations, no indications of a union of several pieces in lines or seams; it conforms to and copies every peculiarity of angle or surface, and it would be difficult to distinguish it in color or form from a nugget from the mine. For contour fillings it is equally available as annealed gold, as piece can be added to piece as long as it is kept dry, and as it is not elastic its approach to the surface of the tooth is more intimate and exact, and it finishes well, giving a surface like polished, molten gold. But to attain these qualities it re quires great care, peculiar instruments and manipulation, and exemption from moisture, even the breath of the mouth is injurious. Patience is indispensable, not only to condense the surface and the margins, but also to finishing it, and then it is unapproachable in beauty and durability. It is not a material that can be taken up, used with any instrument without experience. Inexperience will be pleased with it at first, and disgusted with it at last, unless there is perserverance and the means of using it properly. It is costly because it is dense, and as the pieces are irregular some care is necessary to give it the desired form, and prepare it for the surface intended, so there need be no waste from the file. In general it is best to use small pieces and condense them thoroughly, and this is absolutely necessary near the surface, as any part the instrument did not hit will roughen, which will give it a pitted appearance, and near the margin might cause a leak. The instruments first designed for it are the best I have ever seen, although some other forms can be used, but the surface and margins require fine points. While I have used much annealed gold this has been my favorite since the first introduction of it, and plenty of fillings twenty and more years old attest its value. These fillings are in almost every class of teeth, for the young and old, the soft and the hard, the approximal and crown, and they have suffered but little from marginal decay. But these old fillings had the advantage of separation of the teeth with the file, on the palatine face, often freely, and sometimes on the front face, if it was necessary to give good margins, care being taken not to deface them. Of late years, since it has been more difficult to overcome scruples as to the file, and a marginal show of gold was frequently wanted, peripheral decay has been more common, as was to be expected, chiefly on the median line near the gum, the pre-existing cause of decay asserting itself there as the most exposed point. It matters little what the material is—a mixed metal capable of generating galvanism of itself, a platinum band around the tooth, or an approximal filling that restores contact, anything that is brought close to the enamel and kept there for a time will cause decay. Separation is the only safety, and some of the mouths that I look back to with the greatest pride and satisfaction, are those that I determinately demanded the extraction of teeth that I could have easily filled, to save the rest. Having removed them, some of the others were wedged apart, the continuity of contact was broken and the hold of disease.

The older class of dentists, who made many partial plates, attached by clasps, have, no doubt, a vivid recollection of the trouble they gave in causing the decay of the teeth. They were usually the soundest and strongest teeth in the mouth, wholly without blemish, and in some instances they required filling in six months, or, at longer intervals to be often repeated, until the surface covered by the clasp became banded with fillings, or the tooth failed. As the clasps were well fitted to the teeth the tenderness and decay was not caused by friction and abrasion, but by proximity of surface. In some cases where there was friction the surface became polished and the clasp finally wore into the tooth, sometimes to the depth of its thickness, the constant friction in this way antagonized decay. Then galvanism was held responsible for the decay of clasped teeth, as it is now for the teeth having imperfect fillings. But as all of the teeth having decay were often removed before the insertion of the plate, it was difficult to find a positive pole, until it was held that galvanism was generated by the mixture in the solder, but as in a fine gold plate there was little alloy with no free base metal to be acted on, the reason was not very convincing. As I used nearly the same quality of gold for solder and plate, and habitually, after it was finished, put it into hot nitric acid, or into the battery and coated it with fine gold, there was no waste—nothing for oxygen to attack, and yet, the teeth melted away within such clasps as rapidly as they did in those of silver or other inferior metal—an inequality of product quite out of proportion with the capacity to produce if galvanism was the cause.

It would seem to be unnecessary to invoke the presence of galvanism in the mouth, to account for marginal decay when the presence of the plate, or of a number of fillings prove a pre-existing tendency, that the mere filling of the teeth could not destroy, but could only divert from its first choice of attack to a second. And as we know that the second choice is generally the base of approximal fillings, which it makes the more readily, as the space is unnecessarily narrowed and confined, it would seem that contour fillings that restored the condition that led to the first choice, as near as possible, are an invitation for it to make the second. And as we know that separation diverts and baffles it, and breaks the chain of its attack, or holds it in abeyance, it would seem to be the part of wisdom to meet it in that way, and by a thorough separation defeat it entirely. There is little likelihood that this will be adopted, and some will dispute the premises, but in the presence of experience and observation, with no great fervor. Custom, self-interest, the desire to make costly and flashy fillings, the disinclination to be the first to change, all will plead against it, and those who have achieved leadership by means of it can not afford to abdicate, to change, as vacillation destroys worship. In noticing at some length the two materials, amalgam and gold, it was in reference to galvanism and, in part, to speak of their capabilities, excellence and defects. In forming a judgment of them we must take into consideration, not always the technical perfection attainable by the few, but that degree of utility within the reach of the majority of living operators, leaving the extremes of excellent or of slovenly operations to those who can enjoy or must endure them. Hitherto the popularity of the material has always been made by the success of an operator. What we want is the best material attainable, measured by a skill that can use it, that is not exceptional. It is useless to say that all can be educated up to the highest standard. The great majority of the community must be satisfied with a high mediocrity, even if they can attain that. We can not eliminate from our ranks even the incapables. Society, the church, friends have more to do in giving practice than skill or good judgment, but if these are united there is great responsibility. But high skill is often like great genius, fitful and flighty; it astonishes us by its excellence and depresses us by its failures. It is the fillings that are made in the inspired moments that are the types, it is the others that bring confusion and doubt, not only as to human possibilities, but also of material. A fine filling is the poetry of dentistry—it delights the eye, and it shows its capabilities and possibilities, but very often it is a costly trifle, a credit to manual dexterity, but a discredit to judgment and common sense. But the true dentist aims at practical excellence in all his operations and in the selection of his materials and instruments. He will have no hobby to ride. If he finds that the mallet is useful at times to give greater density to a filling for a particular purpose, he will know when to use it and where, and when to discard it, and he will select one that gives the proper momentum and impact, and he will not change in rapid succession from wood to ivory, from ivory to lead, and from lead to steel, asserting that each is better than the other, in some important particulars, and then, possibly use each one in succession and indiscriminately in condensing the same filling, to give it the particular excellence of each. A great dentist, whose skill is real, the admiration of the profession has no need of such adventitious aids or outposts to support him, that draw attention from merit to particulars, that give him no strength, and weakens others. Concentration of attention should be directed to essentials, that they may be perceived and comprehended, while expatiation on unessentials is bewildering and depressing; it leads to great confusion and it unsettles much. At the present time there is great need of candid investigation of the value of material for saving the teeth. A few may make good fillings that do not leak, and that do save the teeth with annealed gold, but the many fail. From the present outlook the prospect is not cheering, that they will improve and succeed better in the near future.

In the meantime people are rapidly losing their teeth, and that loss comes generally from an acid. Repeated tests of the water in the mouth made at different times of the day show it to be generally neutral. That was to be expected as the constant flow from the ducts, the expectoration by the men and the swallowing of it by the women, would leave little chance for the acid to remain in the mouth. Even that which was taken at meals is soon beyond the reach of tests, if not immediately neutralized by the ever present alkalies in the food, which taken one day with another greatly exceed the acid taken in a given time. If we add to this what is frequently taken by women during pregnancy and at other times, and what the spinsters, whose sensitive stomachs, suffering from a sympathetic and transferred irritation, require almost daily of calcined magnesia, it will be evident that the mouth will contain little acid during the day and that we must look to the night, during sleep, for the time when the teeth suffer the most. Then the ducts rest and pour into the mouth but little fluid and that concentrated; the month at times is nearly or quite dry and parched, the tongue is coated and there is a disagreeable taste in the mouth on awakening. As soon as consciousness returns, and there is a consciousness of the condition of the mouth, the ducts begin to pour out their fluids and the taste disappears. There is rarely stomatitis to account for this, but there is frequently a slight gastritis or hepotitis, but there is oftener a stomach laboring at digestion, and if it is acid it will affect the teeth. Tobacco is only useful so far as this, that it stimulates the flow of saliva that is rapidly expectorated, and the quid acts as a brush in removing food from the teeth, but as it contains a considerable per cent. of free potassa nitrate, it may produce that polished abrasion of the teeth that is frequently seen. The nitric acid acting on the salts and the potash on the fibers, conjointly make clean work. I have frequently had occasion to burn tobacco stems and a little of the leaf, so damp as not to blaze, in a small furnace. As this was generally done in the evening and in the dark, it afforded a fine display of flashing, hissing coruscations running along the stems and through the mass, that was almost infernal, and at first it was startling. As it was constant and always present it seemed to me like cause and effect, especially as the quid is pressed and ground on the teeth that suffer most in that way. That form of decay that cuts a grove across the necks of the teeth near the gum is frequently accompanied by more or less absorption or waste of the socket. The gum is turgid, and when pressed upon a fluid appears near the neck of the tooth that is unhealthy and seemingly acrid. I have noticed it more in strong, bilious constitutions and have thought mercury may have predisposed to it. But many of those persons use tobacco. Fillings in such places are extra hazardous and amalgam is frequently used.

The risk of marginal decay after filling the teeth of young persons is great, and for that reason there should be separation. But they will usually object to it, and they want contour fillings. After the completion of ossification of the frame at about twenty-two, the danger is less, and after thirty-five it is still more diminished. A filling at an early age that exactly restores loss of tooth substance, however perfect it may be at the margins, can never equal the original condition of the tooth, but if proximity is removed, it approaches more nearly to it, and marginal decay will diminish in proportion to the space between the teeth. Fillings of restoration are generally unsightly, but they are sometimes necessary for particular reasons, but space should always be secured between the teeth if possible. They will not then invite disease, and any defect in them will not be so soon apparent. I think if dentists will reflect a little, and examine the junction of the filling and enamel, at the base between the teeth, always the most difficult, they will find not only imperfection but a condition of uncleanliness and deposit; it may be only of a film that is inviting to disease and is uninviting to galvanism, and that they can dismiss the galvanic theory in the forcible language of ex-Gov. Allen, of this State, applied to another subject as, "a damed barren ideality."

### AMALGAM.

BY GUSTAVUS NORTH, D. D. S., SPRINGVILLE, IOWA.

We seldom read a dental journal of late but what contains an article on "Plastic Fillings," and it seems at times that the profession is running wild on the subject.

Amalgam has its place and should be appreciated by the profession, but the profession should be on guard and not be abused by the plastic substance.

I, at one time, condemned amalgam and considered it an unfit material to be employed in the preservation of the teeth. During this period I substituted tinfoil which proved, after several years of constant use a good material if properly used for certain cavities, and a material that proves superior to amalgam for the preservation of tooth structure. But we have certain classes of teeth that amalgam is preferable to tin where there is friction and where the tooth is frail and delicate and will not stand the pressure of a foil filling. In such cases amalgam has its place.

I have changed in my practice but not to the extent of some. I consider amalgam a valuable material to be used for certain occasions or for large cavities in the posterior part of the mouth, but the material must be properly manipulated or it will prove a failure.

I believe amalgam as a material for filling teeth, has been abused by the profession more than any other one material. A great many are ignorant of the proper mode of manipulating it, and condemn it on account of their ignorance.

As much care should be taken in preparing a cavity for amalgam as any other material. The cavity should be kept dry during the operation as well as the material; and as little mercury used in preparing the amalgam as possible after it is thoroughly mixed or prepared, according to the established rules. The mercury should be squeezed or pressed out of the amalgam by the use of chamois skin and tweezers. As it will be impossible to throw off the required amount of mercury by the pressure of the thumb and finger. The material when ready for use should be in quite a hard state, but should be used before crystalization takes place. The tooth should be carefully filled by using small pieces and well condensing each piece until the operation is completed.

Amalgam hardens by crystalizing. A filling should always be finished and thoroughly burnished after this takes place.

I claim that amalgam is a good useful material, but not superior to gold, only for certain occasions; and I am sorry that some members of the profession are carrying it to an extreme, on account of the younger members of the profession.

I believe that the hand that holds the instrument and the brain that guides and instructs the hand, has fully as much to do with the preservation of the teeth as the material employed.

Let each material have its place, and do not be persuaded to abandon any, that has stood the test for many years.

